# One-dimensional confinement and width-dependent bandgap formation in epitaxial graphene nanoribbons

**DOI:** 10.1038/s41467-020-19051-x

**Published:** 2020-12-11

**Authors:** Hrag Karakachian, T. T. Nhung Nguyen, Johannes Aprojanz, Alexei A. Zakharov, Rositsa Yakimova, Philipp Rosenzweig, Craig M. Polley, Thiagarajan Balasubramanian, Christoph Tegenkamp, Stephen R. Power, Ulrich Starke

**Affiliations:** 1 Max-Planck-Institut für Festkörperforschung, Heisenbergstraße 1, 70569 Stuttgart, Germany; 2 Institut für Physik, Technische Universität Chemnitz, Reichenhainer Straße 70, 09126 Chemnitz, Germany; 3 Institut für Festkörperphysik, Leibniz Universität Hannover, Appelstraße 2, 030167 Hannover, Germany; 4 MAX IV Laboratory, Lund University, Fotongatan 2, 22484 Lund, Sweden; 5 IFM, Linköping University, 58183 Linköping, Sweden; 6 School of Physics, Trinity College Dublin, Dublin 2, Ireland

**Keywords:** Surfaces, interfaces and thin films, Electronic devices, Electronic properties and devices, Electronic properties and materials

## Abstract

The ability to define an off state in logic electronics is the key ingredient that is impossible to fulfill using a conventional pristine graphene layer, due to the absence of an electronic bandgap. For years, this property has been the missing element for incorporating graphene into next-generation field effect transistors. In this work, we grow high-quality armchair graphene nanoribbons on the sidewalls of 6H-SiC mesa structures. Angle-resolved photoelectron spectroscopy (ARPES) and scanning tunneling spectroscopy measurements reveal the development of a width-dependent semiconducting gap driven by quantum confinement effects. Furthermore, ARPES demonstrates an ideal one-dimensional electronic behavior that is realized in a graphene-based environment, consisting of well-resolved subbands, dispersing and non-dispersing along and across the ribbons respectively. Our experimental findings, coupled with theoretical tight-binding calculations, set the grounds for a deeper exploration of quantum confinement phenomena and may open intriguing avenues for new low-power electronics.

## Abstract (web summary)

Here, the authors investigate armchair graphene nanoribbons by angle-resolved photoelectron spectroscopy, and show the development of a width-dependent semiconducting gap driven by quantum confinement effects, and an ideal one-dimensional electronic behaviour.

## Introduction

Graphene nanoribbons (GNRs) are considered to be the fundamental building blocks for future carbon-based nanoelectronics. The functionality of GNRs is governed by the detailed atomic structure of their edges^1,2^. Namely, GNRs terminated by zigzag edges demonstrate one-dimensional (1D) single-channel ballistic transport at room temperature^3–5^, while those with armchair edges promise a width-dependent semiconducting behavior^6–11^. In the view of digital applications, the potential of graphene is limited by the lack of a sizeable bandgap in its electronic structure. Hence, the scalable growth of high-quality armchair GNRs (AGNRs) is essential for the development of future graphene-based technologies. Earlier attempts to produce GNRs involved the patterning of pristine graphene layers using standard lithographic means. However, such top-down approaches severely suffer from the lack of atomic-scale precision, resulting in the formation of disordered and rough edges^12–14^. In contrast, the surface-assisted bottom-up approach, for instance the self-assembly of molecular precursors on gold surfaces, was demonstrated to create high-quality GNRs^15–17^. The drawback of this method lies in its use of a metallic substrate, and thus its impracticality to be incorporated in a technologically relevant application. In this respect, the production of GNRs on a semiconducting substrate such as SiC is an attractive avenue^18–21^. Particularly, the selective growth of AGNRs on the sidewalls of SiC mesa structures, is shown to produce well-defined edge morphologies^8,21^ and to provide a better control on the overall ribbon widths as compared to the growth of AGNRs on the $(000\bar 1)$, i.e., the so-called C-face of SiC^19^. Therefore, epitaxial sidewall AGNRs stand as promising candidates for the realization of new tunneling-based field effect concepts^22^, since going beyond conventional metal-oxide-semiconductor field-effect transistor (MOSFET) architectures, almost defect-free semiconductors are mandatory.

In this work, high-quality AGNRs are formed through the periodic modulation of a graphene sheet grown epitaxially on the sidewalls of 6H-SiC mesa structures. Angle-resolved photoelectron spectroscopy (ARPES) reveals peculiar features that differ from previous GNR band structure measurements^23–25^. A combination of well-resolved dispersing and non-dispersing subbands, parallel and perpendicular to the ribbon direction respectively, is observed. In fact, a characteristic band structure topology, resembling a triangular prism as expected for 1D confined Dirac electrons, was never realized thus far in a graphene-based environment. Tight-binding (TB) simulations reveal that the detailed subbands measured in ARPES originate from a mixture of metallic and semiconducting AGNRs, indicating the development of a width-dependent bandgap. This is further corroborated by scanning tunneling spectroscopy (STS) measurements, which confirm that individual ribbons can display either metallic or semiconducting behavior.

## Results

### Epitaxial growth of armchair graphene nanoribbons

**Fig. 1:**
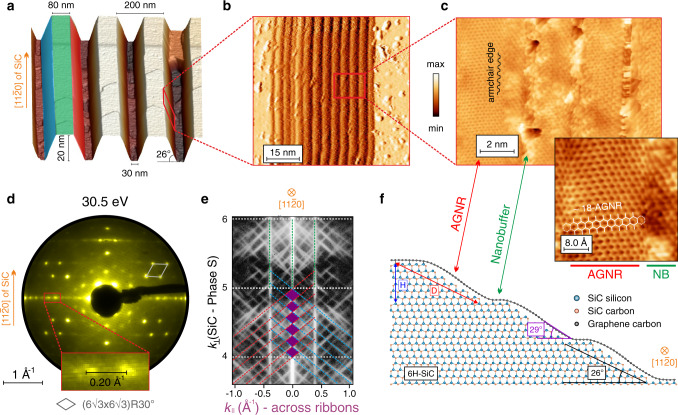
Structural properties of AGNRs epitaxially grown on 6H-SiC sidewalls. **a** Perspective AFM view of the mesa structures with a periodicity of 200 nm. The trench depth is 20 nm and the facet inclination is around (26 ± 2)°. **b** First derivative of a STM topography image taken on a single facet displaying the “ladder structure” (*V* = 0.15 V, *I* = 1.75 nA). **c** Magnified view of the mini-facets highlighting the armchair orientation of the sidewall ribbons (*V* = 2 V, *I* = 0.5 nA). The step edges are not atomically resolved since the vicinal surface is electronically inhomogeneous, due to the presence of AGNRs and nanobuffer (NB) regions having completely different electronic characters. The inset shows a high-resolution topography image taken on a single ribbon with a well-defined armchair edge at the AGNR-nanobuffer border. The AGNR has a width of about 2 nm equivalent to *N* ~ 18 dimers. **d** LEED pattern at *E* = 30.5 eV showing a clear spot-chain oriented perpendicular to the [11$\bar 2$0]-direction. The diffraction spots belonging to the spot-chain have a periodicity of (0.20 ± 0.02) Å^−1^. The gray diamond indicates the $\left( {6\sqrt 3 \times 6\sqrt 3 } \right)\mathrm{R}30^\circ$ superstructure. **e** Reciprocal lattice rods measured by SPA-LEED. The six purple diamond crossings within one full phase-shift of ${\Delta} S = 1$ imply a step height of six SiC bilayers separating two neighboring mini-terraces. **f** Schematic structural model of graphene epitaxially grown on a 6H-SiC sidewall, displaying the formation of free-standing AGNRs and insulating nanobuffer regions.

Graphene on 6H-SiC develops in a well-defined epitaxial relationship with respect to the substrate^26^. The armchair edge of graphene is naturally oriented parallel to the [11$\bar 2$0]-direction of SiC. Accordingly, AGNRs are grown on the sidewalls of 6H-SiC mesa structures, deliberately constructed along the [11$\bar 2$0]-direction as shown in the 3D representation of the atomic force microscopy (AFM) measurement in Fig. 1a. At elevated temperatures, when Si sublimation and graphene growth set in, the armchair sidewalls decompose into a number of approximately 3 nm wide mini-facets^21^ with an average inclination of (26 ± 2)°. This periodic array of mini-facets, which is somewhat reminiscent of a “ladder structure”, can be observed in the scanning tunneling microscopy (STM) image of Fig. 1b. A magnified view of the latter (Fig. 1c) demonstrates AGNRs with high crystallographic quality. The ribbons do not break as they roll over the microstep edges but rather exhibit corrugation due to their strong interaction with the substrate. Using the notation *N*-AGNR to denote a ribbon with *N* dimers across its width, we find ribbons composed of *N* ~ 18 dimers, corresponding to a width of about 2 nm as shown in the inset of Fig. 1c. After graphene growth, the sample is finally covered by about 10^5^ AGNRs/mm, a density high enough to allow the use of integrating experimental techniques such as low-energy electron diffraction (LEED) and ARPES.

Figure 1d represents the LEED pattern of the sample measured after GNR growth. The diamond-like spot arrangement indicated by the gray lines is a consequence of the well-known $\left( {6\sqrt 3 \times 6\sqrt 3 } \right)\mathrm{R}30^\circ$ symmetry of the carbon buffer layer with respect to the basal plane of 6H-SiC^26^. The periodic array of mini-facets constituting the SiC sidewall (as previously seen in STM, Fig. 1b), acts as a diffraction grating for the scattered electrons, which leads to the splitting of diffraction spots and the formation of a spot-chain oriented perpendicular to the [11$\bar 2$0]-direction. The separation between two neighboring spots belonging to the spot-chain amounts to (0.20 ± 0.02) Å^−1^ as shown in the inset of Fig. 1d. This value is equivalent to a periodicity of (3.1 ± 0.3) nm in real space, which agrees well with the period of the “ladder structure” observed in STM. Fig. 1e displays a spot profile analysis LEED (SPA-LEED) image, which is essentially a measure of the reciprocal lattice Ewald rods^27,28^. The vertical basal rods originate from the planar green surface (see Fig. 1a) while the inclined red and blue rods originate from their respective, oppositely inclined red and blue facets. The momentum perpendicular to the sample surface (*k*_⊥_) is described in units of the SiC phase *S*, which is defined as the phase difference between two electron waves scattered from adjacent terraces separated by a step height of a single SiC bilayer. Assuming that the facets consist of mini-steps equivalent to a single SiC bilayer, we would expect the facet rods to intersect the basal rods only at in-phase conditions, i.e., at integer values of *S*. However, in Fig. 1e we observe six crossings (6 purple diamonds) within one full phase-shift of ${\Delta} S = 1$, indicating the presence of mini-terraces separated by steps of full unit-cell height, i.e., six SiC bilayers.

Based on the properties revealed by AFM, STM, LEED, and SPA-LEED, a structural model is proposed for AGNRs in Fig. 1f. A single graphene carpet covers the SiC sidewall where its electronic structure is periodically modulated as it rolls over the microsteps. On the horizontal mini-terraces, the graphene sheet behaves as an insulating nanobuffer layer due to its strong interaction with the substrate. The Si atoms of the topmost SiC layer covalently bond to graphene, saturating its *p*_*z*_ orbitals and preventing the development of a Dirac cone. However, on the vicinal planes, the strong graphene-SiC interaction is lifted due to the different surface registry of the substrate as compared to the basal plane. Consequently, free-standing AGNRs are developed, generating 1D confined Dirac electrons. The mini-facets themselves are unit-cell high (*H* = 1.5 nm) and have a periodicity of *D* = (3.1 ± 0.3) nm. The free-standing ribbons covering the mini-facets are approximately 2 nm wide with an average inclination of (29 ± 1)° relative to the basal plane, as estimated from the ARPES measurements discussed below.

### Electronic structure of armchair graphene nanoribbons

**Fig. 2:**
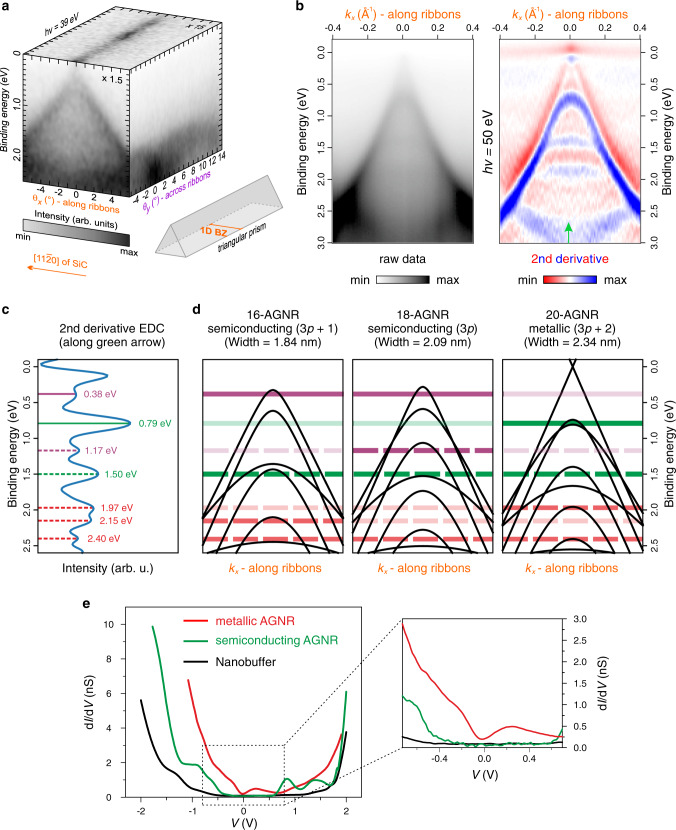
Electronic structure of 1D confined AGNRs. **a** 3D photoelectron intensity distribution $I\left( {E,\theta _x,\theta _y} \right)$ mapping the electronic band structure of AGNRs. The contrasts of the energy-momentum cut along the ribbons and the Fermi surface are enhanced with respect to the cut across the ribbons. The triangular prism sketch represents the overall shape of the electronic structure in a 2D momentum space. The 1D Brillouin zone (BZ) of AGNRs is oriented parallel to the [11$\bar 2$0]-direction of SiC (i.e., along the ribbons). **b** High-resolution ARPES energy-momentum cut taken along the ribbons using a photon energy of 50 eV and its second derivative plot along the energy axis. **c** Second derivative of the energy distribution curve (EDC) taken along the green arrow in (**b**) highlighting the binding energies of the distinct subbands. **d** The electronic structure of AGNRs calculated for different widths using a TB formulation. The colored lines indicate the binding energies of the subbands extracted from (**c**). They have a width equivalent to a binding energy window of 60 meV, representing the error range introduced by the second derivative operation. **e** STS spectra measured in the center of the ribbons of different widths displaying both semiconducting and metallic behaviors. The inset shows a zoom-in around *E*_F_.

An overview of the electronic band structure of AGNRs is depicted in Fig. 2a. Due to the lateral confinement of graphene, the Dirac electrons disperse only in the ribbon direction ($\theta _x$), giving rise to a Fermi surface that consists strictly of a straight line in the perpendicular direction ($\theta _y$). For this reason, in a two-dimensional (2D) momentum space, the electronic structure upholds a triangular prism shape as shown in the sketch of Fig. 2a, whose dispersion (along $\theta _x$) is acquired by projecting that of 2D graphene onto the 1D Brillouin zone (BZ) of the ribbon^1^. Fig. 2b shows a high-resolution ARPES energy-momentum cut taken at normal emission, parallel to the ribbon direction, together with its second derivative along the energy axis. A linear gapless band, characteristic of Dirac fermions in graphene, is observed, and a number of dispersing subbands, striking signatures of 1D quantum confinement, are clearly distinguished at different binding energies. Away from the Dirac cone, a diffuse and nondispersive surface state originating from the $\left( {6\sqrt 3 \times 6\sqrt 3 } \right)\mathrm{R}30^\circ$ reconstruction^29^, is also made visible near the Fermi level (*E*_F_). The Dirac point is situated at *E*_F_, which corresponds to electronically neutral AGNRs. The subband energies are determined by locating the positions of the peak maxima in the second derivative of the energy distribution curve (EDC) taken at $k_x = 0.0\;{\mathrm{{\AA}}}^{ - 1}$ (Fig. 2c). It is important to note that the second derivative operation is ideal for improving the direct visualization of dispersive features, however, it introduces an uncertainty of about 60 meV (i.e., ± 30 meV) on the derived binding energy values. This uncertainty range is estimated by comparing the position of the most prominent subband peak located at 0.82 eV in the raw data, and shifted to 0.79 eV in the second derivative plot. In order to understand the experimentally observed features, the electronic band structure is calculated using a nearest-neighbor TB approach. Previous studies have already shown that both semiconducting and metallic behaviors can be expected when varying the width of AGNRs^1,6^. In particular, ribbons with *N* = 3*p* + 2 (*p* being an integer value) are predicted to be metallic (or display a very small bandgap), whereas all other widths result in semiconducting ribbons whose bandgaps are inversely proportional to their widths^6^. Accordingly, we consider in Fig. 2d the band structures of *N*-AGNRs with *N* = 16, 18, and 20, whose widths are in the 2 nm range suggested by our STM data (the band structures of the intermediate 17- and 19-AGNRs are shown in Supplementary Fig. 2 and are consistent with the TB analysis discussed below). The metallic 20-AGNR appears to give the best fit to the prominent peaks in Fig. 2c. This ribbon shows the linear band, together with subbands at the peaks denoted by solid and dashed green lines. However, the more subtle peaks, indicated by solid and dashed purple lines, are not fully consistent with a metallic ribbon. Instead, these are in excellent agreement with the expected positions of the first and second subbands of a semiconducting 18-AGNR. The subbands of the 16-AGNR on the other hand, provide an excellent fit to the subtle peak located at (0.38 ± 0.03) eV and the more prominent peak at (1.50 ± 0.03) eV. Finally, the lowest lying peaks (marked by red dashed lines), are consistent with subbands from both ribbon types. Our TB analysis suggests that the subband features observed in the ARPES spectrum originate from a superposition of bands from semiconducting and metallic ribbons of slightly different widths. To confirm this hypothesis, we examine STS spectra taken on individual ribbons of varying widths in the range of 2 nm as previously shown in STM. The distinct dI/dV curves in Fig. 2e reveal a ribbon of metallic character (red spectrum) as well as a semiconducting AGNR with a valence band maximum at about 0.35 eV relative to *E*_F_ (green spectrum), consistent with the band structures from ARPES and TB. A wide-bandgap nanobuffer layer is also identified in Fig. 2e (black spectrum) which acts as an insulating barrier separating two neighboring ribbons. Thus, the spectroscopic measurements, coupled with the theoretical band structure calculations, provide clear evidence of an ideal 1D electronic system in a graphene-based environment, where both metallic and semiconducting behaviors can be realized. Based on the results obtained in STS, ARPES and TB simulations (and additional STM/STS measurements shown in Supplementary Fig. 3), the actual bandgap (separating the valence band from the conduction band) of the semiconducting AGNRs is found to be around (0.75 ± 0.05) eV.

**Fig. 3:**
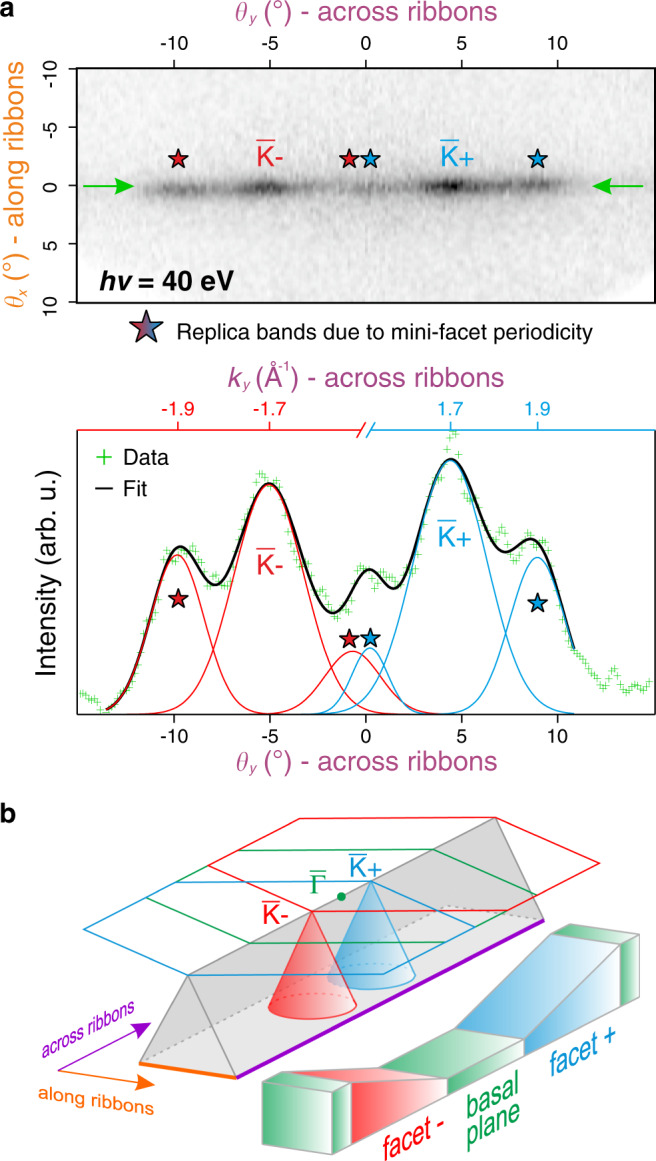
Fermi surface. **a** High-resolution Fermi surface map acquired at a photon energy of 40 eV, and a MDC taken at $\theta _x = 0^\circ$ (indicated by the green arrows). The Dirac cones of the oppositely oriented parasitic graphene layers are observed at their respective $\overline {\mathrm{K}}$-points ($\overline {\mathrm{K}}_ -$ and $\overline {\mathrm{K}}_ +$). Replica bands are marked with star signs. The conversion to *k*-space is made separately for each parasitic sidewall graphene layer relative to its own normal emission. **b** Schematic representing the electronic structure of AGNRs (i.e., the triangular prism) on top of which the 2D BZs and the Dirac cones of the parasitic sidewall graphene layers are superimposed (grown on *facet−* and *facet+*, respectively). The BZ of the parasitic graphene layer overgrown on the basal plane is also shown (green hexagon).

Figure 3a shows a high-resolution Fermi surface map and the corresponding momentum distribution curve (MDC) taken at $\theta _x = 0^\circ$. The straight line in the Fermi surface that extends across the ribbons throughout the entire probed *k*-space is a result of 1D confinement as discussed above. Such characteristic 1D behaviors have been examined before in other systems, e.g., using self-organized metallic nanowires on semiconductor surfaces^30,31^. The intensity modulation observed in the Fermi surface is mainly due to an undesired parasitic graphene layer that, in small patches, has overgrown the SiC sidewalls and has an estimated surface area coverage on the order of a few percent. A 2D graphene sheet that is grown on an inclined facet would correspond to a 2D hexagonal BZ shifted away from normal emission relative to the basal plane as sketched in Fig. 3b (the amount by which the BZ is shifted in reciprocal space is strictly related to the angle of inclination of the sidewall graphene in real space^32^). At a photon energy of 40 eV and a spectrometer work function of 4.3 eV as used in the experiment, the $\overline {\mathrm{K}}$-point of graphene would appear at ±34° with respect to its own normal emission. This means, that the two most intense points in the Fermi surface located at −5.1° and 4.4° (as determined by the MDC fit of Fig. 3a), simply represent the $\overline {\mathrm{K}}$-points ($\overline {\mathrm{K}}_ -$ and $\overline {\mathrm{K}}_ +$) of the oppositely inclined parasitic sidewall graphene layers having an average inclination of (29 ± 1)°. The remaining intense points marked by the star signs appear at a distance of (0.20 ± 0.02) Å^−1^ from their associated $\overline {\mathrm{K}}$-points, which agrees well with the periodicity observed in LEED and STM (see Fig. 1). Therefore, these intensity enhancements represent the replica bands of the parasitic graphene sheets, resulting from diffraction by their underlying periodic structures, i.e., the mini-facets. A more detailed discussion concerning the intensity modulations observed in ARPES can be found in the Supplementary Information (Supplementary Fig. 1).

**Fig. 4:**
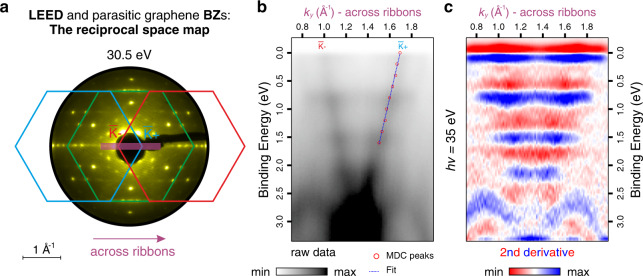
Electronic structure perpendicular to the ribbon direction. **a** Reciprocal space map for a clear orientation in *k*-space. **b** ARPES energy-momentum cut taken across the ribbons at normal emission relative to the basal plane. The dispersing bands represent the Dirac cones of the oppositely inclined parasitic graphene layers. The *k*-space conversion is made relative to the normal emission of the parasitic graphene layer grown on the blue facet (i.e., blue hexagon in the RSM). The red circles indicate the momentum distribution curve (MDC) peak positions at different binding energies and the blue dotted line shows a linear fit to those MDC peaks. **c** Second derivative plot of (**b**) along the energy axis where nondispersive subbands of AGNRs are clearly observed, demonstrating a characteristic behavior of 1D confinement.

In Fig. 4, the electronic properties are examined across the ribbons. In order to clearly indicate the orientation in *k*-space, we display the 2D reciprocal space map (RSM), which is constructed by drawing the BZs of the parasitic sidewall graphene layers on top of the LEED pattern (Fig. 4a). A high-resolution ARPES energy-momentum cut is taken along the purple line shown in the RSM (i.e., perpendicular to the ribbon direction). The strongly dispersing bands in Fig. 4b arise from the non-confined parasitic graphene layers overgrown on the two oppositely inclined facets, which were used to calibrate the phase space of the probed system. Only a single branch of the Dirac cone is visible in the spectrum for each sidewall graphene due to the well-known dark corridor effect^33^. By fitting a linear curve to the band courses of the parasitic layers, a Fermi velocity of *v*_F_ ~ 1.2 × 10^6^ m/s is extracted, which is in good agreement with the Fermi velocity of a pristine graphene layer found in literature^34,35^. In the second derivative along the energy axis (Fig. 4c), we find AGNR subbands located at the same binding energies as the ones observed in Fig. 2c. However, the lack of dispersion across the ribbons as opposed to their dispersive nature along the ribbons (Fig. 2c) clearly highlights the 1D nature of the epitaxially grown sidewall AGNRs. Finally, we note that the features observed below 2.5 eV binding energy constitute the valence band of the 6H-SiC substrate.

## Discussion

We have demonstrated that high-quality AGNRs are formed through the periodic modulation of the electronic structure of graphene when epitaxially grown on the sidewalls of 6H-SiC mesa structures. Although the atomic structure and in particular the hexagonal symmetry of the graphene carpet rolling over the SiC microsteps is conserved, the electronic structure periodically changes character due to its different modes of interaction with the substrate. On the vicinal surfaces, the absence of a covalent bonding between the graphene layer and the substrate leads to the formation of free-standing AGNRs, generating 1D confined Dirac electrons. In contrast, on the horizontal mini-terraces, the strong graphene-SiC interaction produces electronically inactive nanobuffer regions, which act as insulating barriers separating two neighboring ribbons and providing electronically defined edges. The electronic structure of the system is investigated using ARPES and STS. A Fermi surface consisting strictly of a straight line is detected, which is indicative of the 1D character of the probed AGNRs. Distinct sets of subbands that arise from quantum confinement are also well resolved. By reproducing the experimentally observed dispersive features using a TB formulation, we clearly identify the development of a width-dependent bandgap. The graphene growth results in the formation of AGNRs of different widths, displaying either semiconducting or metallic behavior, which in their turn are individually measured using a local STS probe. Our results demonstrate the remarkable functionalization of graphene’s electronic properties via 1D quantum confinement. For specific AGNR widths, an average bandgap of about (0.75 ± 0.05) eV is introduced in the electronic structure, which is desirable for defining an *off* state in digital electronics. The production of almost defect-free and narrow AGNRs with smooth edges, on a technologically viable substrate such as SiC, will allow the realization of new transistor concepts, e.g., tunneling field effect transistors, which are expected to reveal lower subthreshold slopes compared to conventional MOSFETs. These concepts were proven recently using electrostatically doped GNRs^22^. Therefore, the epitaxially grown AGNRs post-processed further by intercalation^36–40^, may open the avenue for next-generation low-power electronics.

## Methods

### Sample preparation

Nominally on-axis, single crystalline, *n*-doped 6H-SiC(0001) wafer pieces (purchased from SiCrystal GmbH) were used as substrates for the AGNR growth. They were atomically flattened via hydrogen etching, which was carried out in an inductively heated reactor equipped with a graphite susceptor^29,39^. Mesa structures with a lateral dimension of about 150 nm and a height of 20 nm were defined using a combination of e-beam lithography and reactive ion etching (gas mixture 20/7 SF_6_/O_2_, power 30 W, pressure 0.05 mbar). The selective growth of AGNRs was achieved by annealing the samples in an inductively heated furnace at 1800 °C under 850 mbar of argon atmosphere for 1 min^21^.

### ARPES measurements

ARPES measurements were carried out at the Bloch beamline of the MAX IV synchrotron facility in Lund, Sweden. High-resolution energy-momentum cuts were measured using a high performance deflector-based DA30 hemispherical analyzer from ScientaOmicron. The energy and angular resolutions were set to 15 meV and 0.1° respectively. The spot-size of the beam measured 10 × 24 µm^2^, simultaneously probing around 2000 AGNRs. All ARPES data were acquired in ultra-high vacuum (UHV) at a sample temperature of 80 K. Additional, preliminary ARPES measurements (not shown in the manuscript) were conducted at the 1^2^ endstation of the UE112 beamline at BESSY II, Helmholtz-Zentrum Berlin.

### STM/STS measurements

The STM/STS measurements were performed in UHV (*p* < 2 × 10^−11^ mbar) at 80 K using a commercial Omicron LT-STM. The sample was degassed at an elevated temperature of 800 K for several hours before being transferred to the low-temperature chamber. Several electrochemically etched tungsten tips were utilized during the experiments. A lock-in detection technique was used to accumulate and average over 50 STS spectra taken on each individual ribbon.

### Tight-binding model

The electronic band structures of AGNRs were calculated using a nearest-neighbor (NN) tight-binding (TB) Hamiltonian

1 $$H = t\mathop {\sum }\limits_{ < i,j > } \hat c_i^\dagger \hat c_j$$

where $\hat c_i^\dagger$and $\hat c_j$ are the creation and annihilation operators, *i*, *j* are atomic site indices and the sum is restricted to neighboring sites only. In this model, an AGNR with *N* *=* *3p* *+* *2* is metallic, whereas other ribbons have bandgaps that scale inversely with *N*, and which are proportional to *t*, the NN hopping parameter^6^. We set $t = - 3.0$ eV in our simulations, and note that this single-parameter model captures the appearance and approximate size of the bandgap in semiconducting AGNRs, and also the subband structure which occurs in both types of ribbons. A more precise quantitative fit could be achieved by introducing additional terms in the Hamiltonian which slightly modify the bandgap and/or shift the relative subband positions. The role of a number of such terms is discussed in the Supplementary Information (Supplementary note 2).

## Supplementary information

Supplementary Information

## Data Availability

The authors confirm that all relevant data supporting the findings of this study are included in the article and its Supplementary Information files. Additional data are available from the corresponding author upon request.

## Code availability

The procedures used to process and evaluate the experimental data, as well as compute the tight-binding band structures are available from the corresponding author upon request.
