# Supplementary material for: One-dimensional confinement and width-dependent bandgap formation in epitaxial graphene nanoribbons
Source: Nat Commun. 2020 Dec 11;11:6380. doi: 10.1038/s41467-020-19051-x (PMC7733518; doi:10.1038/s41467-020-19051-x)
Supplement: Supplementary file 1 — Supplementary Information [file 41467_2020_19051_MOESM1_ESM.pdf]

Supplementary Information: One-dimensional confinement and width-dependent bandgap formation in epitaxial graphene nanoribbons

Hrag Karakachian[1*], T. T. Nhung Nguyen[2], Johannes Aprojanz[2, 3], Alexei A. Zakharov[4], Rositsa Yakimova[5], Philipp Rosenzweig[1], Craig M. Polley[4], Thiagarajan Balasubramanian[4], Christoph Tegenkamp[2, 3], Stephen R. Power[6] & Ulrich Starke[1]

[1] Max-Planck-Institut für Festkörperforschung, Heisenbergstraße 1, 70569 Stuttgart, Germany
[2] Institut für Physik, Technische Universität Chemnitz, Reichenhainer Straße 70, 09126, Chemnitz, Germany
[3] Institut für Festkörperphysik, Leibniz Universität Hannover, Appelstraße 2, 030167 Hannover, Germany
[4] MAX IV Laboratory, Lund University, Fotongatan 2, 22484 Lund, Sweden
[5] IFM, Linköping University, 58183 Linköping, Sweden
[6] School of Physics, Trinity College Dublin, Dublin 2, Ireland

Supplementary note 1: Intensity enhancements in the Fermi surfaces

Supplementary Figure 1 displays a series of Fermi surfaces measured at different photon energies together with their respective momentum distribution curves (MDCs) taken at $\theta_x = 0°$. Similarly to Fig. 3 in the manuscript, the MDCs are fitted by using six Gaussian peaks, two of which represent the $\bar{K}$-points ($\bar{K}_-$ and $\bar{K}_+$) of the parasitic graphene layers overgrown on the oppositely inclined facets, as shown by the reciprocal space maps (RSMs) drawn at the bottom of Supplementary Figure 1. The remaining four peaks marked by the star symbols represent the first-order replica bands of the Dirac cones located at $\bar{K}_-$ (two red stars) and $\bar{K}_+$ (two blue stars) that result from diffraction by the periodic array of mini-facets constituting the SiC sidewalls. The positions of the individual Gaussian peaks are determined from the basic photoemission equation

$$\theta_y = \pm\left[\sin^{-1}\left(\frac{\hbar k_y}{\sqrt{2m_e(h\nu - \varphi)}}\right) - \delta\right] \tag{1}$$

where θ_y is the emission angle across the ribbons and δ is the average inclination of the parasitic graphene layer on the facet. At different photon energies, $\bar{K}_-$ and $\bar{K}_+$ are found at θ_y values corresponding to $|k_y|$ = 1.7 Å^{-1} (as expected for the $\bar{K}$-point of graphene) and δ = (29 ± 1)°. The positions of the replica bands on the other hand correspond to $|k_y|$ = 1.9 Å^{-1} and 1.5 Å^{-1}, which are in excellent agreement with the (0.20 ± 0.02) Å^{-1} periodicity found in LEED (Fig. 1d in manuscript). A similar replica structure has been observed in nanorippled graphene monolayers grown on 6H-SiC macrofacets[1]. It is important to note that the parasitic graphene layers on the facets are the main, but not the only, contributors to the intensity modulations observed in the Fermi surfaces. Previous ARPES studies on 1D electronic systems have shown that the photoelectron intensity of a non-dispersing subband (such as the one present in the Fermi surface) may vary as a function of emission angle due to optical transition matrix element effects[2,3] as well as experimental geometric conditions[4].

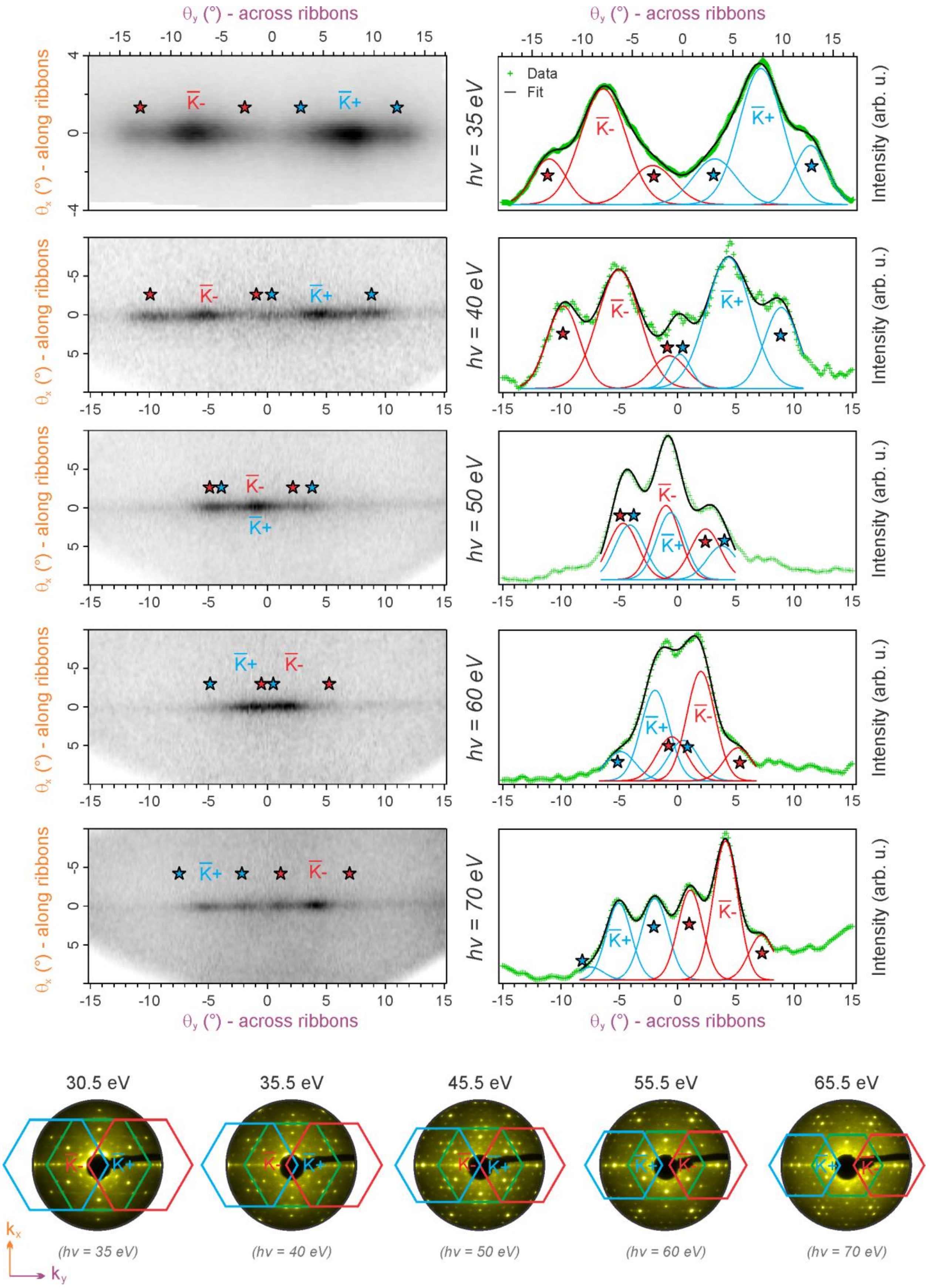

θy (°) - across ribbons
θx (°) - along ribbons
Data
Fit
Intensity (arb. u.)
hν = 35 eV
hν = 40 eV
hν = 50 eV
hν = 60 eV
hν = 70 eV
K̄-
K̄+
30.5 eV
35.5 eV
45.5 eV
55.5 eV
65.5 eV
(hν = 35 eV)
(hν = 40 eV)
(hν = 50 eV)
(hν = 60 eV)
(hν = 70 eV)
kx
ky

Supplementary Figure 1 – A series of Fermi surfaces measured at different photon energies together with their respective MDCs taken at $\theta_x = 0°$. The $\overline{K}$-points of the parasitic graphene layers overgrown on the oppositely inclined facets are termed $\overline{K}_-$ and $\overline{K}_+$. The star signs represent the first-order replica bands of the Dirac cones located at $\overline{K}_-$ and $\overline{K}_+$. The RSMs at different energies (corresponding to the different photon energies minus the spectrometer work function) are also displayed.

Supplementary note 2: Tight-binding simulations

In the simulations shown in Fig. 2d of the main paper, we use a nearest-neighbor TB Hamiltonian with a single parameter, $t = -3.0$ eV, and note an excellent qualitative agreement with our experimental results. In particular, prominent features from ARPES measurements are in excellent agreement with the expected confinement-induced subbands from AGNRs of the appropriate width. Furthermore, since the ARPES technique samples a large number of nanoribbons, the signal is a superposition of subbands from a range of ribbons of slightly different widths. In the first row of Supplementary Figure 2 (panels a-e), we reproduce the band structures of the 16-, 18- and 20-AGNRs from the main text, together with 17- and 19-AGNRs. The energies of prominent ARPES features, shown by horizontal green, purple and red lines, all coincide with the onset of AGNR subbands.

In this note, we explore the robustness of the band structures when additional terms are added to the Hamiltonian either to include higher-order hopping terms or to simulate various perturbations that may arise in realistic nanoribbons.

Firstly, we note that a range of values from approximately -2.6 to -3.2 eV are reported in the literature for *t*, depending on how the fitting is performed, the type of *ab initio* or experimental data used for comparison, and whether graphite, graphene or multi-layer graphene is considered[5-7]. The value of *t* may also differ slightly between suspended and epitaxial graphene. However, bandgaps and subband onsets are proportional to *t*, so rescaling this parameter does not qualitatively change the band structure.

In Supplementary Figure 2, we show the band structures of a metallic (middle row, panels f-j) and a semiconducting (bottom row, panels k-o) AGNR for a range of more complicated scenarios. From left to right, we compare band structures for the following:

- The standard NN model (panels f, k).
- A model also including 2nd and 3rd nearest-neighbor hopping terms (panels g, l), with values -0.074|t| and -0.067|t| respectively[8].
- A NN model with a non-uniform strain profile (panels h, m), to account for a variation in bond lengths between the centre and the edge of the ribbon. In this case, we illustrate the effect using a simple Gaussian height profile $h(y) = a\, e^{-\frac{(y-b)^2}{c^2}}$, with a = 0.10 nm, b =

ribbon centre, c = 0.74 nm. This leads to a variation in nearest-neighbour bond-lengths throughout the ribbon, which we account for in our model by changing the hopping parameters accordingly[9].

- A NN model with an on-site potential V= -0.4|t| ~ -1 eV applied to the edge sites (panels i, n), to mimic edge-termination effects. Similar qualitative results are found for a positive potential.
- A NN model with edge disorder in the form of 5% of edge sites being removed (panels j, o). Band structures were calculated for a 40 nm (200 unit cell) periodic supercell, and a Brillouin zone unfolding procedure[10-12] was carried out to produce an effective band structure in the conventional Brillouin zone.

In each case, the bands are shown re-centred so that the Dirac point or bandgap centre lies at *E* = 0. Each of the terms above introduces slight modifications to the subband structure, but the qualitative picture observed for the simplest 1NN model is robust.

One significant effect of some models is the opening of a very small bandgap in metallic ribbons. *Ab initio* calculations show that electron-electron interactions can cause zigzag-edged GNRs to lose their metallicity due to a spin-splitting of edge states. This effect can be captured by including a simple mean-field Hubbard term in the TB Hamiltonian[13]. While *ab* initio results also show a small gap opening for metallic AGNRs[14], a similar Hubbard term is not sufficient to replicate the effect in TB models. Instead, a number of competing mechanisms are available, including edge deformation[14], higher-order hopping terms[15] and non-local electron-electron interactions[16]. Indeed, each of the perturbations considered in Supplementary Figure 2, if sufficiently strong, can open a small gap.

From the above, it is not necessarily clear which mechanism is at play when gap opening in metallic AGNRs is observed in experiment. Furthermore, some experiments[17] appear not to find the expected trend for *N* = 3p + 2 ribbons with gap opening, and instead show the zero gap behaviour predicted by a simple TB model. Other studies, meanwhile, do show gap opening for 3p + 2 ribbons[18]. This suggests that the lifting of metallic behaviour may not be a general feature in all ribbons, but is particular to the ribbon environment and can be affected by factors such as substrate, strain and edge termination. Our experimental results do not show a clear feature of gap formation and suggest that we have true metallic ribbons, justifying the use of a NN TB model. The absence of a clear gap further highlights the pristine nature of our ribbons, as significant perturbations tend to lead to gap formation.

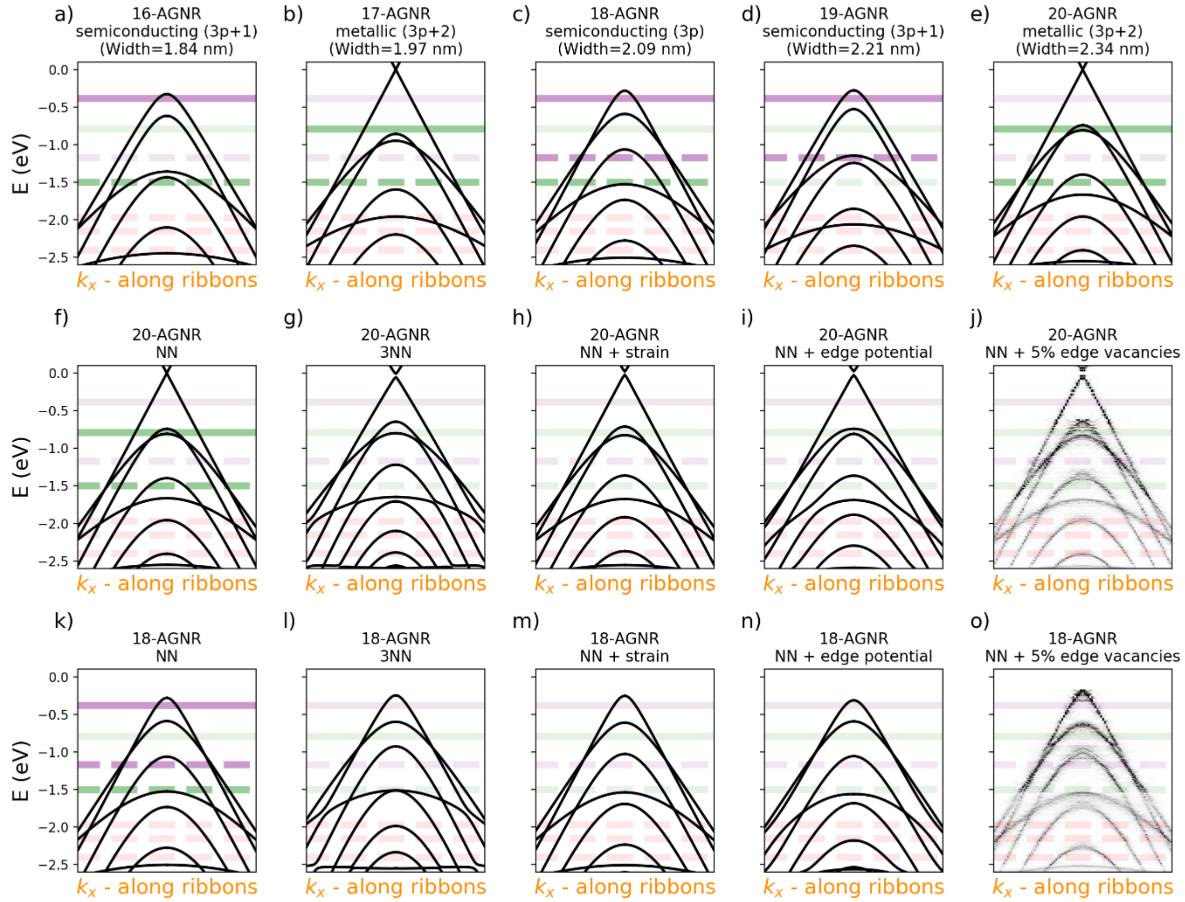

Supplementary Figure 2 – Electronic band structure of AGNRs calculated for **a-e**, different ribbon widths using a nearest-neighbor TB Hamiltonian **f-j**, metallic 20-AGNR with additional terms in the Hamiltonian **k-o**, semiconducting 18-AGNR with additional terms in the Hamiltonian.

Supplementary note 3: STM/STS on semiconducting AGNRs of different widths

Supplementary Figure 3 presents STM/STS measurements taken on individual AGNRs of slightly different widths. The number of dimer lines across the ribbons are estimated to be about $N \sim 16$ and $N \sim 18$. Even though atomically resolved STM images are obtained, the *exact* number of N (representing the electronically active part of the ribbon) can be determined only if the ‘first’ and ‘last’ free-standing carbon atoms (that are not bonded to the underlying Si atoms of the SiC substrate) at the edges of the ribbons are specifically identified. Since AGNRs and nanobuffer layers are both composed of carbon atoms, it is not possible to precisely identify the onset of the ribbon at the AGNR/nanobuffer border. STS measurements reveal an inverse proportionality between the sizes of the electronic gaps and the ribbon widths as predicted by tight-binding calculations. The thinner ribbon (AGNR 1) shows a semiconducting gap of about 0.8 eV, while that of the wider ribbon (AGNR 2) is about 0.7 eV. Therefore, the growth procedure results in the

production of ~ 2 nm wide AGNRs, the semiconducting ones of which exhibit an average bandgap value of (0.75 ± 0.05) eV, desirable for next-generation tunneling field-effect transistor designs.

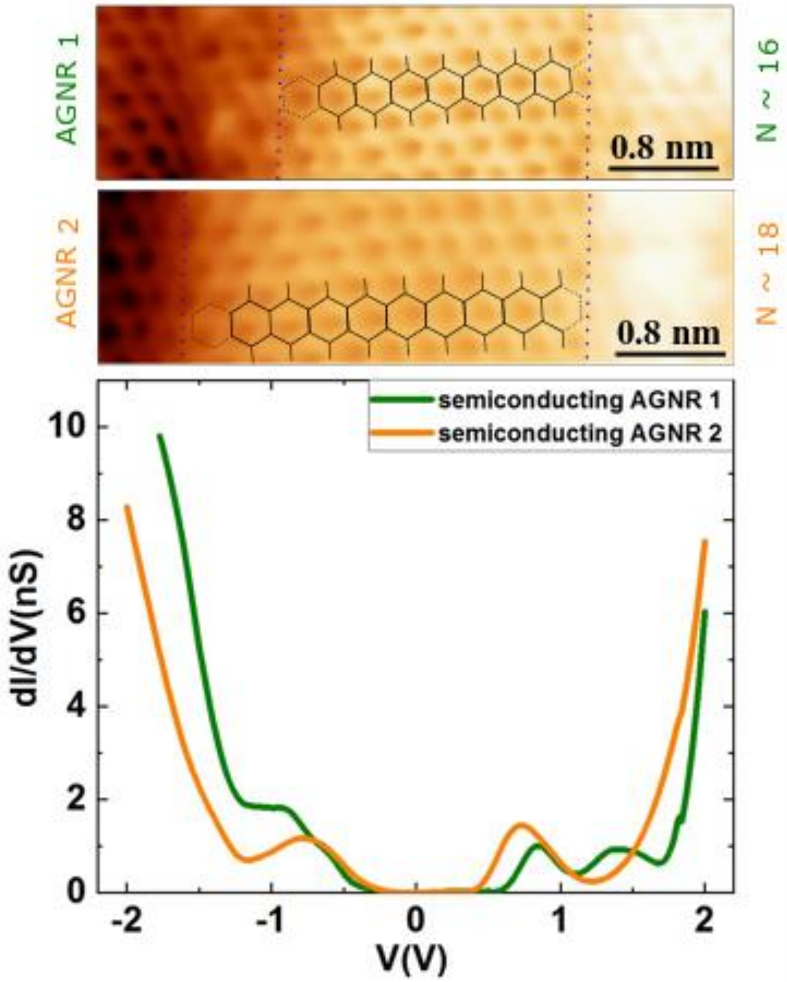

Supplementary Figure 3 – STM high-resolution topography images (V = 2 V, I = 0.5 nA) and STS spectra taken on semiconducting AGNRs of slightly different widths. AGNR 1 and AGNR 2 are *N ~ 16* and *N ~ 18* dimers wide, respectively. The associated bandgap values are about 0.8 eV and 0.7 eV. The STS spectra are measured at the center of the ribbons.

Supplementary references

1. Ienaga, K., Iimori, T., Yaji, K., Miyamachi, T., Nakashima, S., Takahashi, Y., Fukuma, K., Hayashi, S., Kajiwara, T., Visikovskiy, A., Mase, K., Nakatsuji, K., Tanaka, S. & Komori, F. Modulation of electron-phonon coupling in one-dimensionally nanorippled graphene on a macrofacet of 6H-SiC. *Nano Lett.* **17**, 3527 (2017).

2. Meyer, S., Schäfer, J., Blumenstein, C., Höpfner, P., Bostwick, A., McChesney, J. L., Rotenberg, E. & Claessen, R. Strictly one-dimensional electron system in Au chains on Ge (001) revealed by k-space mapping. *Phys. Rev. B* **83**, 121411(R) (2011).
3. Yaji, K., Mochizuki, I., Kim, S., Takeichi, Y., Harasawa, A., Ohtsubo, Y., Le Fèvre, P., Bertran, F., Taleb-Ibrahimi, A., Kakizaki, A. & Komori, F. Fermi gas behavior of a one-dimensional metallic band of Pt-induced nanowires on Ge (001). *Phys. Rev. B* **87**, 241413(R) (2013).
4. Senkovskiy, B. V., Usachov, D. Y., Fedorov, A. V., Haberer, D., Ehlen, N., Fischer, F. R. & Grüneis, A. Finding the hidden valence band of N = 7 armchair graphene nanoribbons with angle-resolved photoemission spectroscopy. *2D Mater.* **5**, 035007 (2018).
5. Charlier, J. C., Gonze, X. & Michenaud, J. P. First-principles study of the electronic properties of graphite. *Phys. Rev. B* **43**, 4579 (1991).
6. Reich, S., Maultzsch, J., Thomsen, C. & Ordejon, P. Tight-binding description of graphene. *Phys. Rev. B* **66**, 035412 (2002).
7. Kuzmenko, A. B., Crassee, I., van der Marel, D., Blake, P. & Novoselov, K. S. Determination of the gate-tunable band gap and tight-binding parameters in bilayer graphene using infrared spectroscopy. *Phys. Rev. B* **80**, 165406 (2009).
8. Hancock, Y., Upptsu, A., Saloriutta, K., Harju, A. & Puska, M. J. Generalized tight-binding transport model for graphene nanoribbon-based systems. *Phys. Rev. B* **81**, 245402 (2010).
9. Pereira, V. M., Castro Neto, A. H. & Peres, N. M. R. *Phys. Rev. B* **80**, 045401 (2009).
10. Boykin, T. B. & Klimeck, G. Practical application of zone-folding concepts in tight-binding calculations. *Phys. Rev. B* **71**, 115215 (2005).
11. Deretzis, I., Calogero, G., Angilella, G. G. N. & La Magna, A. Role of basis sets on the unfolding of supercell band structures: from tight-binding to density functional theory. *EPL* **107**, 27006 (2014).
12. Farjam, M. Visualizing the influence of point defects on the electronic band structure of graphene. *J. Phys.: Condens. Matter* **26**, 155502 (2014).
13. Fujita, M., Wakabayashi, K., Nakada, K. & Kusakabe, K. Peculiar localized state at zigzag graphite edge. *J. Phys. Soc. Jpn.* **65**, 1920 (1996).
14. Son, Y., Cohen, M. L. & Louie, S. G. Energy gaps in graphene nanoribbons. *Phys. Rev. Lett.* **97**, 216803 (2006).
15. Gunlycke, D. & White, C. T. Tight-binding energy dispersions of armchair-edge graphene nanostrips. *Phys. Rev. B* **77**, 115116 (2008).
16. Hadipour, H., Şaşıoğlu, E., Bagherpour, F., Friedrich, C., Blügel, S. & Mertig, I. Screening of long-range Coulomb interaction in graphene nanoribbons: armchair versus zigzag edges. *Phys. Rev. B* **98**, 205123 (2018).
17. Magda, G. Z., Jin, X., Hagyimási, I., Vancsó, P., Osváth, Z., Nemes-Incze, P., Hwang, C., Biró, L. P. & Tapasztó, L. Room-temperature magnetic order on zigzag edges of narrow graphene nanoribbons. *Nature* **514**, 608 (2014).
18. Wang, W., Zhou, M., Li, X., Li, S., Wu, X., Duan, W. & He, L. Energy gaps of atomically precise graphene sidewall nanoribbons. *Phys. Rev. B* **93**, 241403(R) (2016).
